# An examination of the impact of online feedback and social distance on the implicit self-identity of adolescents: behavioral and ERP evidence

**DOI:** 10.1093/scan/nsag024

**Published:** 2026-06-10

**Authors:** Xiaoyu Wang, Wei Fan

**Affiliations:** Cognition and Human Behavior Key Laboratory of Hunan Province, School of Education Science, Hunan Normal University, Changsha 410081, China; Institute of Interdisciplinary Studies, Hunan Normal University, Changsha 410081, China; Cognition and Human Behavior Key Laboratory of Hunan Province, School of Education Science, Hunan Normal University, Changsha 410081, China; Institute of Interdisciplinary Studies, Hunan Normal University, Changsha 410081, China

**Keywords:** social media, online feedback, social distance, self-identity, adolescents

## Abstract

Adolescents’ self-identity is shaped by online feedback’s valence (positive/negative) and social distance (near/far). This study tested their interactive effects on implicit self-identity and neural correlates. Experiment 1 used an implicit association measure (D-score) to assess self-identity across ages. Participants received near (peer) or far (stranger) positive/negative feedback. Distant positive feedback reduced D-scores (weaker self-identity links) versus near positive feedback; negative feedback showed the opposite. Late adolescents had higher D-scores for near versus far feedback—indicating heightened sensitivity to proximal social cues during identity formation. Experiment 2 employed ERPs to explore neural mechanisms. Distant positive feedback elicited faster P3s (prioritized processing of socially distant positives). For near feedback, positive versus negative stimuli showed smaller early LPP amplitudes—reflecting differential motivational attention. These findings reveal that feedback valence and social distance jointly modulate implicit self-identity. Neural markers (P3/LPP) uncover distinct processing priorities for proximal/distal positive/negative feedback—highlighting the interplay of social context and neural mechanisms in adolescent self-development.

## Introduction

Adolescence is a critical period for forming a stable, integrated self-identity (Zhou and Guo 2006; drawing on Erikson 1968). Traditional questionnaire methods are limited in assessing the implicit, automatic aspects of this construct due to introspective bounds and social desirability (Bargh 1994). Researchers therefore employ implicit measures, such as the well-established Implicit Association Test (IAT; Greenwald *et al*. 2022) and Go/No-go Association Tasks (GNATs). These tools are effective in tapping into implicit self-evaluations in adolescent samples (e.g. decreasing implicit self-esteem across adolescence; Cai *et al*. 2014) and have been applied to study domain-specific self-identities, such as in sports (Pluta *et al*. 2025) and drinking behaviors (Foster *et al*. 2014).

Social media is a critical environment for adolescent self-identity development. In China, over 200 million adolescents are active users (CNNIC 2025). Adolescents’ self-identity is closely linked to their engagement on these platforms, with explicit self-identification and evaluation predicting actual usage (Cathelyn *et al*. 2022). Online feedback (e.g. likes, comments) provides constant social evaluation that shapes self-concept, particularly when it comes from peers (Lee *et al*. 2014). While positive feedback enhances interaction and support (Vogel *et al*. 2014), excessive exposure can fragment self-concept (Israelashvili *et al.* 2012). Social distance moderates this impact: peer feedback, perceived as in-group evaluation, fosters greater receptivity (Tajfel and Turner 1979, Xu and Xie 2011).

A key unresolved question is how and when evaluative feedback from peers shapes the adolescent self at an implicit, automatic level. To address this, we employ the GNAT to operationalize implicit self-identity—defined as the automatic association strength between the self-concept and positive attributes.

We hypothesize (H1): Adolescents’ implicit self-identity is more strongly influenced by near-source feedback than far-source. Near positive feedback enhances implicit self-identity; near negative feedback may have stronger effects, with outcomes varying by age.

Neuroscientific evidence underscores the plausibility of these effects. A core self-referential brain network (medial prefrontal cortex (mPFC), anterior cingulate cortex (ACC), posterior cingulate cortex (PCC)) supports self-continuity (Heatherton *et al*. 2004, Northoff 2017) and is engaged by social media interactions (Crone and Konijn 2018), with feedback-checking linked to longitudinal brain changes in adolescents (Maza *et al*. 2023). Event-related potentials (ERPs) are ideal for capturing the millisecond dynamics of this feedback processing. Components like the P3 and late positive potential (LPP) index stimulus salience and motivational relevance, with self-relevant and valenced stimuli modulating their amplitudes (Miyakoshi *et al*. 2007, Chen *et al*. 2011, Gyurak and Etkin 2014, Chen *et al*. 2023). Furthermore, positive versus negative self-information differentially modulates the LPP and N400, reflecting motivated and emotional evaluation (Herbert *et al*. 2011, Fields and Kuperberg 2015).

This leads to our neural hypothesis (H2): Positive (vs negative) social feedback will elicit a larger P3 and a reduced LPP, indicating heightened attentional capture and differential motivated processing. This neural differentiation will be amplified for feedback from near (vs far) social sources.

This study employs two experiments to investigate how the valence and source of social feedback influence implicit self-identity and its underlying neural mechanisms among adolescents of different ages. The findings will elucidate the cognitive and neural mechanisms through which social feedback shapes self-identity in digital environments, thereby deepening our understanding of digital identity formation.

## Experiment 1

Experiment 1 examined the effects of online feedback and social distance on adolescents’ implicit self-identity across age stages. We hypothesized the following: (i) there would be a significant interaction between social distance and age, with levels of implicit self-identity varying by age and source proximity and (ii) there would be a significant interaction between social distance and feedback valence, with feedback from closer social sources exerting a stronger influence on self-identity.

### Methods

#### Participants

Participants were recruited from local schools and online communities. Sample size was determined using G*Power 3.1 (Faul *et al*. 2007) for a three-factor mixed design, requiring a minimum of 63 participants (effect size *d *= 0.5, *α*  =  0.05, power(*1-β)* = 0.8). Ultimately, 146 adolescents participated: early adolescents: 50 (28 male, 22 female; *M*_age_ = 13.67 ± 0.67 years), mid-adolescents: 50 (25 male, 25 female; *M*_age_ = 15.17 ± 0.38 years), and late adolescents: 46 (16 male, 30 female; *M*_age_ = 18.47 ± 0.90 years). Each participant received monetary compensation after completing the experiment.

#### Materials and stimuli

##### GNAT task stimuli

Self/non-self words:Self: “I”, “we”, “own”, “self”, “myself”; Non-Self: “they”, “his”, “others”, “outsiders”. Attribute words selection: see online Supplementary Material.

Self-Identity Scale (SIS; Ochse and Plug 1986; α = 0.727): **1**9 items, 4-point scale (e.g. “I have clear life goals”). For further details, see online Supplementary Material.

Inclusion of Other in the Self (IOS) Scale (Aron *et al*. 1992): 7 overlapping circles (1 = separate, 7 = overlapping) to measure social distance. For further details, see online Supplementary Material.

Feedback stimuli: (i) Simulated WeChat Moments: Photoshop-generated posts with “likes” (counts) and comments (likelihood). Selected for positivity variance (Li *et al*. 2011, Tan 2013). (ii) Feedback sources: System 1: Age-matched, remote volunteers (out-of-school peers). System 2: University peers (on-campus proximity).

#### Procedure

The experiment employed a 3 (adolescent age: early/middle/late) × 2 (feedback valence: positive/negative) × 2 (social distance: near/far) mixed design (age: between-subjects; feedback valence/distance: within-subjects), with GNAT D-score as the dependent variable.

Participants first completed social media usage and self-identity questionnaires (mirroring Experiment 2), then uploaded selfies/captions via QR code to simulate WeChat Moments. Their posts were published on two simulated systems: System 1 (remote age-matched volunteers) and System 2 (campus peers, near distance). The researchers explained to participants that the volunteers in System 1 were recruited from schools nationwide and were your age, making them strangers you didn’t know. In System 2, the volunteers were recruited from their own school, with most being from the same college as you, including your roommates and friends or acquaintances from your major. Four hundred volunteers (8 groups, ≤50/group) provided confidential feedback (likes/comments).

Next, participants completed vocabulary learning and waited for feedback before the GNAT task. The GNAT had two phases: Phase 1 (500 ms fixation → 3 s positive feedback → self-related word responses via space bar) and Phase 2 (after 64 trials, responding to self/non-self words), with tasks and feedback fully counterbalanced.

**Figure 1:**
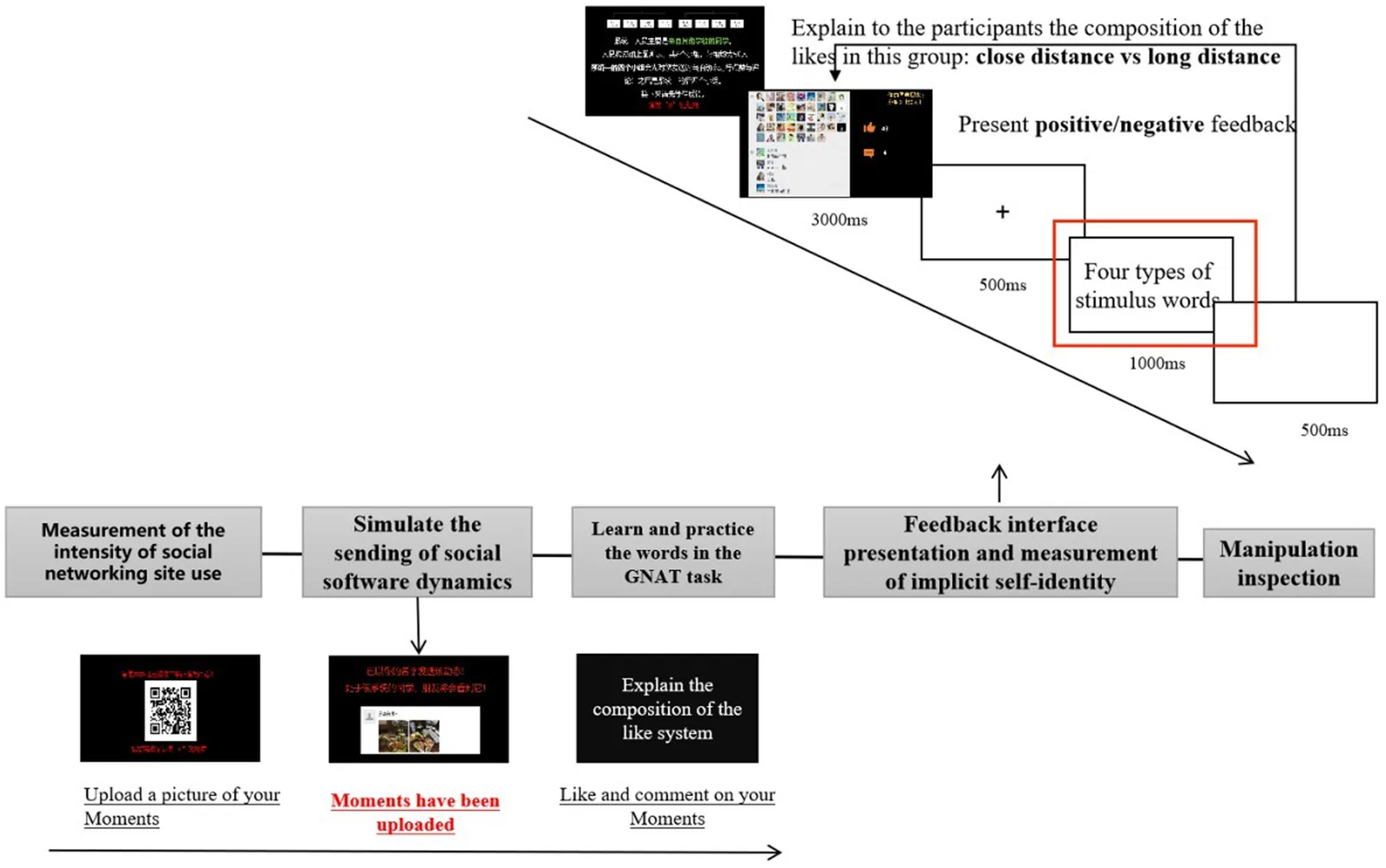
Flowchart outlining the procedure of Experiment 1 and Experiment 2, including key steps: measurement of social networking site use, simulation of social software dynamics, GNAT task practice, feedback interface presentation, implicit self-identity measurement, manipulation check.

Post-task, they completed IOS Scale. System 2 feedback followed the same protocol; all conditions were counterbalanced, and IOS was re-administered. Finally, a manipulation check evaluated platform experience recall, feedback interface realism (7-point), and feedback positivity (7-point). The flowchart is shown in Fig. 1.

#### Data analysis

The D-score, a reaction-time-invariant measure of implicit self-identity with established criterion validity (Teachman 2007) was computed GNAT performance. The calculation involved (i) removing trials with reaction times (RTs) faster than 300 ms, (ii) replacing incorrect trial RTs with a penalty value (the mean correct RT plus 600 ms), (iii) calculating the mean RT for both the self-positive (compatible) and self-negative (incompatible) task blocks, incorporating the adjusted error trials, and (iv) deriving the final D-score by subtracting the mean compatible RT from the mean incompatible RT and dividing the result by the pooled standard deviation of all RTs across both blocks. A higher D-score indicates a stronger automatic association between self-concept and positive attributes. Behavioral data were collected via E-Prime 2.0 and analyzed in SPSS 23.0 using a three-factor repeated-measures ANOVA on D-score across experimental conditions. The specific formula is provided in the appendix.

### Results

#### Manipulation checks

A paired-samples *t*-test on the IOS scale confirmed the successful manipulation of social distance (near vs far). Scores for the far source (*M *= 2.41, *SD *= 0.86) were significantly lower than for the near source (*M *= 6.13, *SD *= 0.57), *t*(145) = 33.244, *p *< 0.001, Cohen’s *d* =*−*5.09.

Another paired-samples *t*-test on the 7-point valence ratings verified the feedback valence (positive vs negative) manipulation. Ratings for positive feedback (*M *= 6.18, *SD *= 0.56) were significantly higher than for negative feedback (*M *= 1.86, *SD *= 0.73), *t*(145) = 42.014, *p *< 0.001, Cohen’s *d *= 6.64.

#### D-score results

A three-factor repeated-measures ANOVA on D-scores (Feedback Valence × Social Distance × Age Stage) was conducted. The main effect of feedback valence was significant *F* (1, 141) = 29.470, *p *< 0. 001, $\eta_{p}^{2}$= 0.173. D-scores were significantly higher under positive feedback (*M *= 0.346 ± 0.035) than under negative feedback (*M *= 0.120 ± 0.032), *p *< 0. 001, 95% CI = [0.144, 0.309]. The main effects of social distance and age stage were not significant (both *p* values > .05).

**Figure 2:**
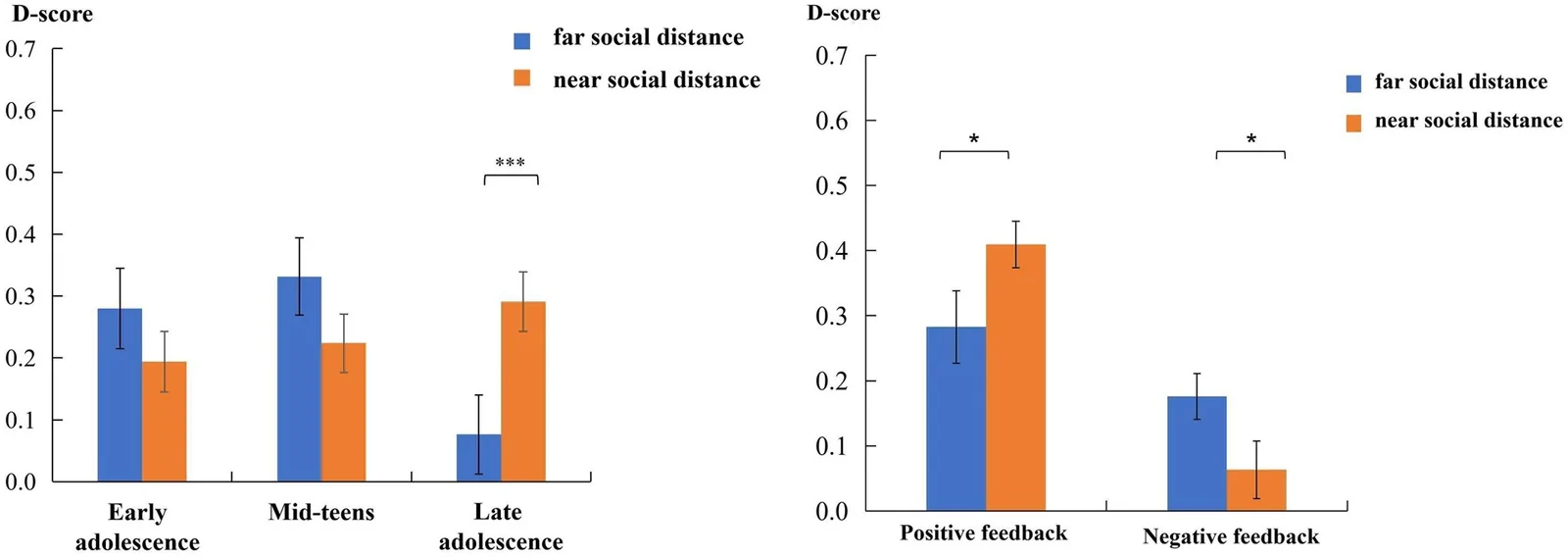
Experiment 1 interaction; error lines indicate standard errors.

Critically, a significant Age Stage × Social Distance interaction emerged, *F* (2, 141) = 7.593, *p *< 0. 001, $\eta_{p}^{2}$= 0.097. Simple effects analysis revealed that this interaction was primarily driven by late adolescents. For this group, D-scores were significantly lower when feedback came from a far source (*M *= 0.076 ± 0.064) compared to a near source (*M *= 0.291 ± 0.048) feedback, *p *< 0. 001, 95% CI = [−0.344, −0.085]; no significant effects were found for early (*p *= 0.202) or middle (*p *= 0.096) adolescents as shown in the left panel of Fig. 2.

The Feedback Valence × Social Distance interaction was also significant, *F* (1, 141) = 9.257, *p *< 0. 05,$\eta_{p}^{2}$ = 0.062. Simple effects analyses showed that for positive feedback, D-scores were lower when from a far (*M *= 0.283 ± 0.056) versus near source (*M *= 0.409 ± 0.036), *p *< 0. 05, with a 95% CI = [−0.249, −0.005]; for negative feedback, D-scores were higher when from a far (*M *= 0.176 ± 0.035) versus near source (*M *= 0.063 ± 0.176), *p *< 0. 05, 95% CI = [0.021, 0.204]; as shown in the right panel of Fig. 2.

The three-way interaction among feedback valence, social distance, and adolescent age was not significant (*p*> .05).

### Discussion

Experiment 1 yielded three principal findings. First, a significant Adolescent Age × Social Distance interaction indicated that the effect of social distance on implicit self-identity (D-scores) was specific to late adolescents, who showed higher D-scores for feedback from near versus far sources. This supports Hypothesis 1 within this age group and aligns with developmental perspectives highlighting late adolescence as a critical period for self-concept differentiation (Meilman 1979), and with work noting age-dependent complexities in how social distance relates to the self (Li 2019).

Second, a significant Feedback Valence × Social Distance interaction revealed that positive feedback enhanced D-scores more when from a near source. This pattern partially supports Hypothesis 2 and is consistent with in-group bias and favoritism (Tajfel and Turner 1979), where in-group affirmation is particularly potent for self-identity reinforcement—a mechanism related to in-group protection (Sedikides 2021).

Third, within the same interaction, negative feedback was more detrimental to D-scores when from a near versus far source. This further corroborates Hypothesis 2, suggesting that negative evaluations from proximal peers pose a greater threat to self-identity. Conversely, the relative discounting of distant negative feedback aligns with strategies such as outgroup derogation to buffer self-esteem (Knobloch-Westerwick 2020).

The absence of a significant three-way interaction (Feedback × Distance × Age) suggests that the basic pattern of how social distance modulates feedback’s impact may be consistent across adolescence. However, its measurable behavioral consequence on implicit self-identity emerged distinctly in late adolescence. This specific result—that the modulation of implicit self-identity by social distance was robust and isolated to late adolescents—provides the direct empirical rationale for Experiment 2. To precisely investigate the neurocognitive mechanisms underlying this behaviorally defined effect, we focused Experiment 2 on this age group, employing ERPs to examine how feedback valence and social distance jointly shape the neural correlates of implicit self-identity.

## Experiment 2

Experiment 2 used the GNAT paradigm and ERPs to explore the neural mechanisms underlying how online feedback valence and social distance affect self-identity formation.

### Methods

#### Participants

Sample size was calculated using G* Power 3.1 (Faul *et al*. 2007) for a two-factor within-subjects design (effect size *d *= 0.4, *α  *=  0.05, power (*1-β)* = 0.8), yielding a minimum of 28 participants. In total, 32 undergraduate students (17–22 years, *M*_age_ = 18.2 ± 0.61, 15 male/17 female) were recruited, with monetary compensation for completing the experiment.

##### Materials

Same as Experiment 1.

##### Design and procedure

This experiment employed a 2 × 2 within-subjects design with feedback valence (positive/negative) and social distance (near/far) as factors. The independent variables were feedback valence and social distance (both within subjects), and the dependent variables included the D-score and ERP components (N1, P300, and LPC). Experimental procedures are the same as that of Experiment 1.

### Data analysis

EEG data were acquired with a 32-channel ERP system (Brain Products, Germany) using the 10-20 international electrode placement. Reference and ground electrodes were placed at FCz and GND, respectively. Data were collected with a 0.01–100 Hz bandpass filter and 1000 Hz sampling rate (impedance <10 kΩ). Preprocessing and analysis used MATLAB R2016b and EEGLAB 14.1.2 (Delorme and Makeig 2004). Offline, data were rereferenced to bilateral mastoid averages, filtered (0.2–30 Hz), and epochs exceeding ±70 µV were rejected. Epochs (−100 to 1000 ms relative to self-identity word onset; Nguyen and Dorjee 2022) were baseline corrected (−100 to 0 ms). Artifactual trials were manually inspected and rejected; eye movements were corrected via ICA. Average ERPs were computed per condition. Electrodes (Fz, Cz, and Pz) were selected based on topography, waveform maps, and prior studies. P3 peaks were detected using a local search algorithm (Luck 2014). Mean amplitudes for N1/P2/N2/P3 were analyzed at Fz/Cz/Pz, respectively. LPP mean amplitudes (early: 400–700 ms; late: 701–1000 ms) were calculated from P3/P4/Pz, following prior work (Hajcak *et al*. 2012, Kujawa, Weinberg, *et al*. 2012, Kujawa *et al*. 2013).

SPSS 23.0 performed repeated-measures ANOVAs: For N1/P2/N2/P3: 2 (feedback valence: positive/negative) × 2 (social distance: near/far) × 3 (electrodes: Cz/Pz/Fz); for LPP (early/late): 2 (feedback valence) × 2 (social distance) on mean amplitudes from P3/P4/Pz.

### Results

#### Behavioral data

##### (i) Manipulation checks

A paired samples *t*-test of the IOS scores for near and far subjects found that the IOS scores for the far group (*M *= 2.31, *SD *= 0.76) were significantly smaller than the IOS scores for the near group (*M *= 5.90, *SD *= 0.44), *t*(30) = 32.21, Cohen’s *d=* −5.78*, p *< 0.001. A paired-samples *t*-test of subjects’ seven-point ratings of the positivity of the feedback they received showed that the positive feedback material scores (*M *= 5.57, *SD *= 0.26) were significantly greater than the negative feedback material scores (*M *= 1.76, *SD *= 0.78), *t*(30) = 41.033, Cohen’s *d *= 6.55, *p *< 0.001.

##### (ii) D-score results

The main effect of feedback potency was significant *F* (1, 31) = 5.497, *p *< 0. 05,$\eta_{p}^{2}$= 0.151. D-scores under positive feedback (*M *= 0.230 ± 0.042) were significantly higher than D-scores under negative feedback (*M *= 0.083 ± 0.047), *p *< 0. 001, 95% CI = [0.019, 0.274]; the main effect of psychological distance was significant *F* (1, 31) = 29.498, *p *< 0. 001,$\eta_{p}^{2}$ = 0.488. D-scores were significantly lower under feedback from a distance (*M *= 0.028 ± 0.036) than under negative feedback (*M *= 0.286 ± 0.043), *p *< 0. 001, 95% CI = [−0.355, −0.161];

The interaction between feedback potency and social distance was significant, *F* (1, 31) = 6.046, *p *< 0. 05,$\eta_{p}^{2}$ = 0.163. A simple effects analysis revealed that individuals’ D-scores were significantly lower when they received positive feedback from a far social distance (*M *= 0.023 ± 0.054) than when they received positive feedback from a near social distance (*M *= 0.437 ± 0.066), *p *< 0. 05, 95% CI = [−0.565, −0.262]; there was no significant difference in D-scores when individuals received negative feedback from a far social distance and when individuals received negative feedback from a near social distance.

#### ERP results

##### N1 average amplitude (100–150 ms)

A 2 (feedback valence: positive vs negative) × 2 (social distance: near vs far) × 3 (electrode site: Fz, Cz, Pz) repeated-measures ANOVA on N1 mean amplitude revealed a significant main effect of social distance, *F* (1, 30) = 9.708, *p < *0.01,$\eta_{p}^{2}$ = 0.244. The mean N1 amplitude in the near social distance condition (*M *= −1.858 ± 0.362) was more negative compared to the mean N1 amplitude in the far social distance condition (*M *= −0.229 ± 0.362), *p *< 0. 01, 95% CI = [−2.697, −0.561]; the main effect of region was significant, *F* (1, 30) = 4.157, *p < *0. 05,$\eta_{p}^{2}$ = 0.122, and by two-by-two comparisons, it was found that the mean N1 amplitude in the Pz region (*M *= −1.678 ± 0.484) was more negative compared to the lower mean N1 amplitude in the Cz region (*M *= −0.773 ± 0.439), *p *< 0.95% CI = [−1.623, −0.187]; the mean N1 amplitude in the Pz region (*M *= −1.678 ± 0.484) was more negative compared to the mean lower N1 amplitude in the Fz region (*M *= −0.7680 ± 0.494), *p *< 0. 05, 95% CI = [−1.976, −0.020].

**Figure 3:**
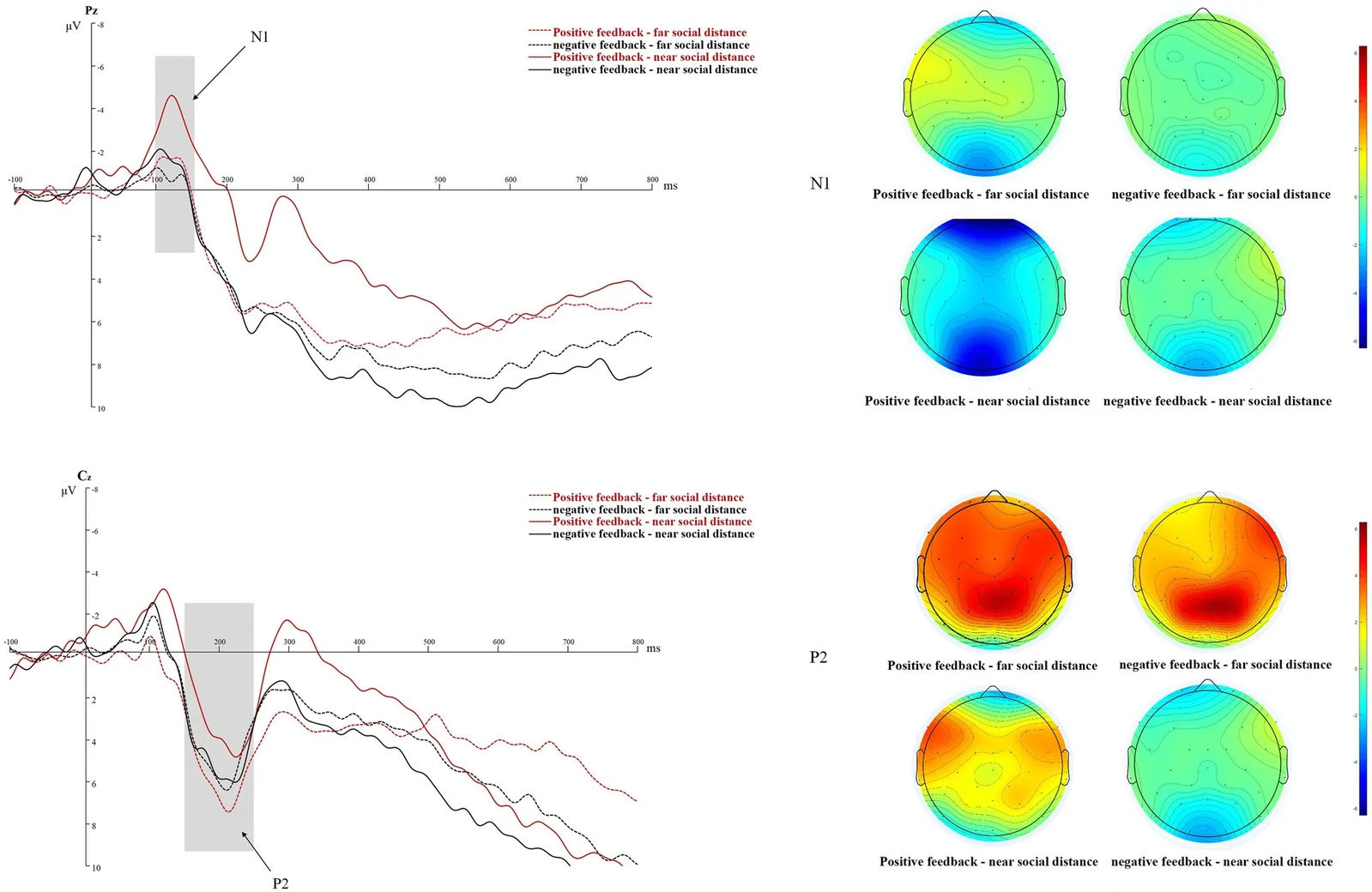
Brain waveforms (left) and brain topography (right of the N1 and P2 components evoked at the Pz and Cz points during decision-making in the four conditions.

A significant Valence × Distance interaction was found, *F* (1, 30) = 6.683, *p *< 0. 05,$\eta_{p}^{2}$ = 0.182. A simple effects analysis revealed that individuals’ mean N1 amplitude in the near-social-distance condition when they received positive feedback (*M *= −2.756 ± 0.777) was more negative when compared to the far-social-distance condition (*M *= −0.068 ± 0.543) was more negative compared to the far-social-distance condition, *p *< 0. 01, 95% CI = [−4.169, −1.207]. Individuals’ N1 mean amplitude in the near-social distance condition (*M *= −0.961 ± 0.560) was not significantly different when they received negative feedback (*M *= −0.390 ± 0.222) compared to the far-social-distance condition, *p *> 0. 05 (see left panel of Fig. 3).

The interaction between region and feedback was significant, *F* (2, 30) = 5.908, *p *< 0. 01,$\eta_{p}^{2}$ = 0.165. A simple effects analysis revealed that in the Pz region, individuals were more negative when they received positive feedback (*M *= −2.454 ± 0.657) compared to the mean N1 amplitude in the receiving negative feedback condition (*M *= −0.902 ± 0.395), *p *< 0. 01, 95% CI = [−2.545, −0.560], no significant differences in other regions.

##### P2 average amplitude (150–250 ms)

The ANOVA revealed a significant main effect of electrode site, *F*(1, 30) = 17.237, *p *< .001, $\eta_{p}^{2}$ = .365. The P2 amplitude at Pz (*M* = 4.90) was larger than at Cz (*M *= 2.45, *p *< .001) and Fz *(M *= 2.88, *p *< .001). The main effect of valence was marginal (*p*= .054) and that of distance was not significant (*p *> .05).

**Figure 4:**
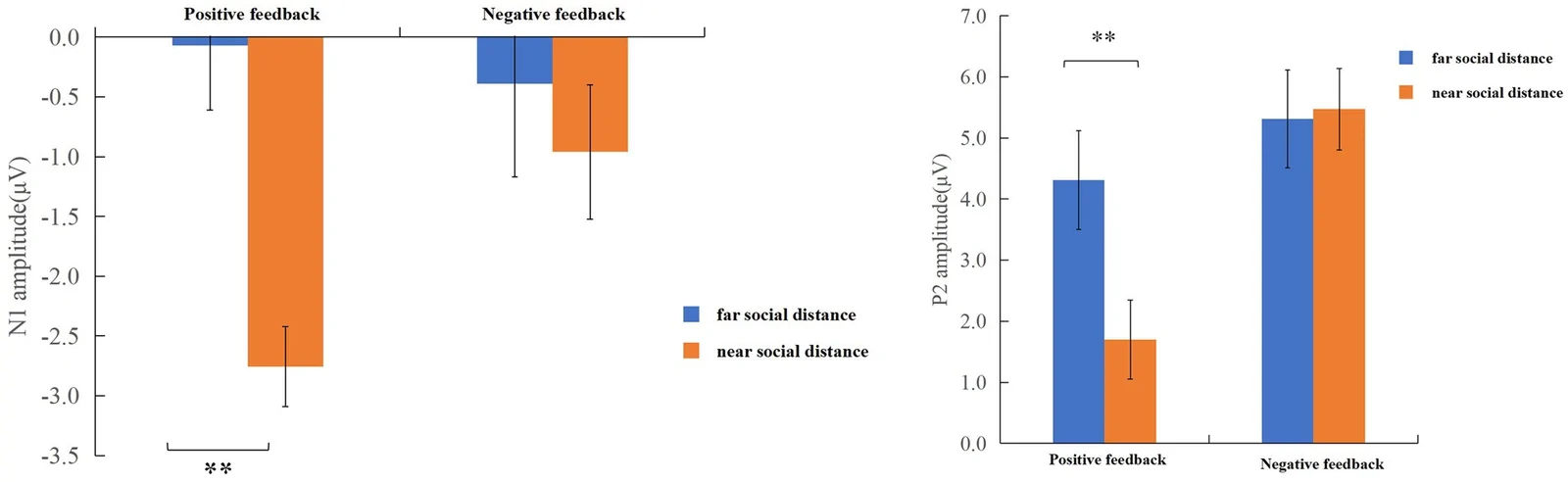
Histograms of the average amplitude of the four conditions N1 (left) and p2 components (right); error lines indicate standard errors.

A significant Valence × Distance interaction was found, *F* (1, 30) = 6.998, *p *< 0. 05,$\eta_{p}^{2}$ = 0.189. Simple effects analysis showed that for positive feedback, the P2 was larger in the far- (*M *= 4.192 ± 0.940) than in the near-distance condition (*M *= 1.564 ± 0.819), *p *< 0. 05, 95% CI = [0.485, 4.773]. For negative feedback, the distance effect was not significant (*p *> .05); see right panel of Fig. 4.

Significant interactions were also found for Electrode Site × Valence, *F* (2, 30) = 15.960, *p *< 0. 001,$\eta_{p}^{2}$ = 0.347 and Electrode Site × Distance, *F* (2, 30) = 3.344, *p *< 0. 05, $\eta_{p}^{2}$= 0.199. Follow-up simple effects analyses indicated that at the Pz site: (i) negative feedback elicited a larger P2 (*M *= 6.281, *SD*= 0.773) than positive feedback (*M *= 3.520, *SD*= 0.810), *p *< .001, 95% CI [1.474, 4.048] and (ii) far-distance feedback elicited a larger P2 (*M *= 5.744, *SD* = 0.857) than near-distance feedback *(M *= 4.057, *SD*= 0.736), *p *< .01, 95% CI [0.329, 3.046]. No such effects were significant at other sites (*p *> .05).

##### N2 average wave amplitude (250–350 ms)

The ANOVA revealed significant main effects of valence, *F* (1, 30) = 7.256, *p *< 0. 05,$\eta_{p}^{2}$ = 0.195, and social distance *F* (1, 30) = 5.615, *p < *0. 05,$\eta_{p}^{2}$ = 0.158. The N2 amplitude was larger (more negative) for negative (*M *= 4.234, *SD* = 0.610) than for positive *(M *= 2.780, *SD* = 0.665) feedback, 95% CI [0.352, 2.566], and for near- (*M *= 2.598, SD = 0.634) than for far-distance (*M *= 4.415, *SD*= 0.749) feedback, *p *< .05, 95% CI [−3.382, −0.525].

A significant Valence × Distance interaction was found, *F* (1, 30) = 14.81, *p *< 0. 01,$\eta_{p}^{2}$ = 0.331. Simple effects analysis showed that for positive feedback, the N2 was larger (more negative) in the far- (*M *= 0.759 ± 0.834) than in the near-distance condition (*M *= 4.801 ± 0.857), *p *< 0. 01, 95% CI = [−6.174, −1.911]. For negative feedback, the distance effect was not significant (*p*> .05). The main effect of electrode site and related interactions were not significant *(p* > .05), see left panel of Figure 6.

##### P3 average wave amplitude (330–430 ms)

The ANOVA revealed a significant main effect of valence, *F* (1, 30) = 7.322, *p *< 0. 05,$\eta_{p}^{2}$ = 0.196. The P3 amplitude was larger for negative (*M *= 5.365 ± 0.736) than for positive (*M *= 3.666 ± 0.742), 95% CI = [0.417, 2.982]. The main effect of distance was not significant (p> .05).

**Figure 5:**
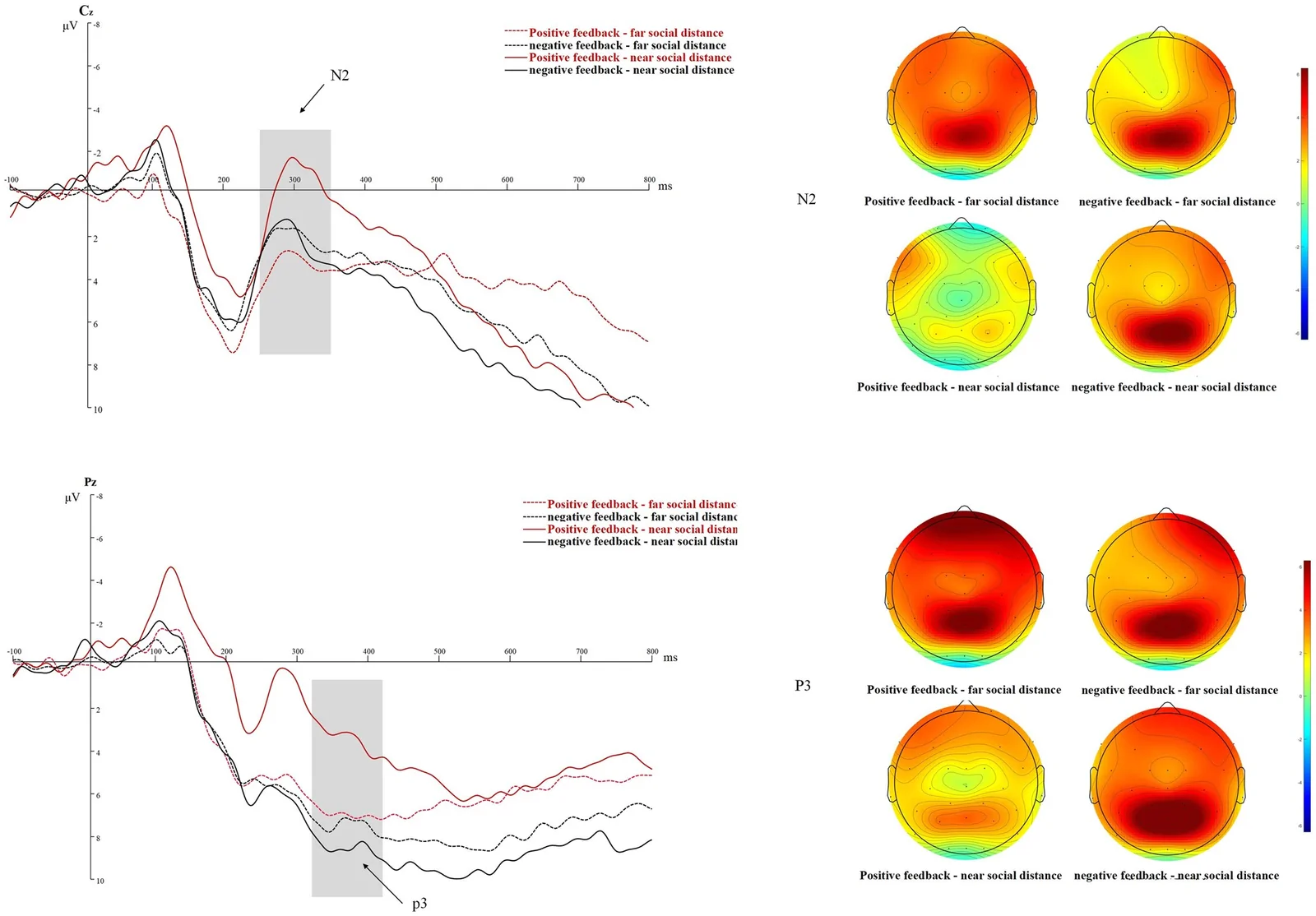
Brain waveforms (left side) and brain topography (right side of N2 and P3 components evoked at the Pz and Cz points during decision making in the four conditions.

A significant Valence × Distance interaction was found, *F* (1, 30) = 6.452, *p *< 0. 05,$\eta_{p}^{2}$ = 0.177. Simple effects analysis showed that for positive feedback, the P3 was larger in the far-(*M *= 4.785 ± 0.812) than in the near-distance condition (*M *= 2.383 ± 0.983), *p *< 0. 05, 95% CI = [0.943, 4.286]. For negative feedback, the distance effect was not significant (*p *> .05); see right panel of Fig. 5. The main effect of electrode site and related interactions were not significant (*p* > .05).

**Figure 6:**
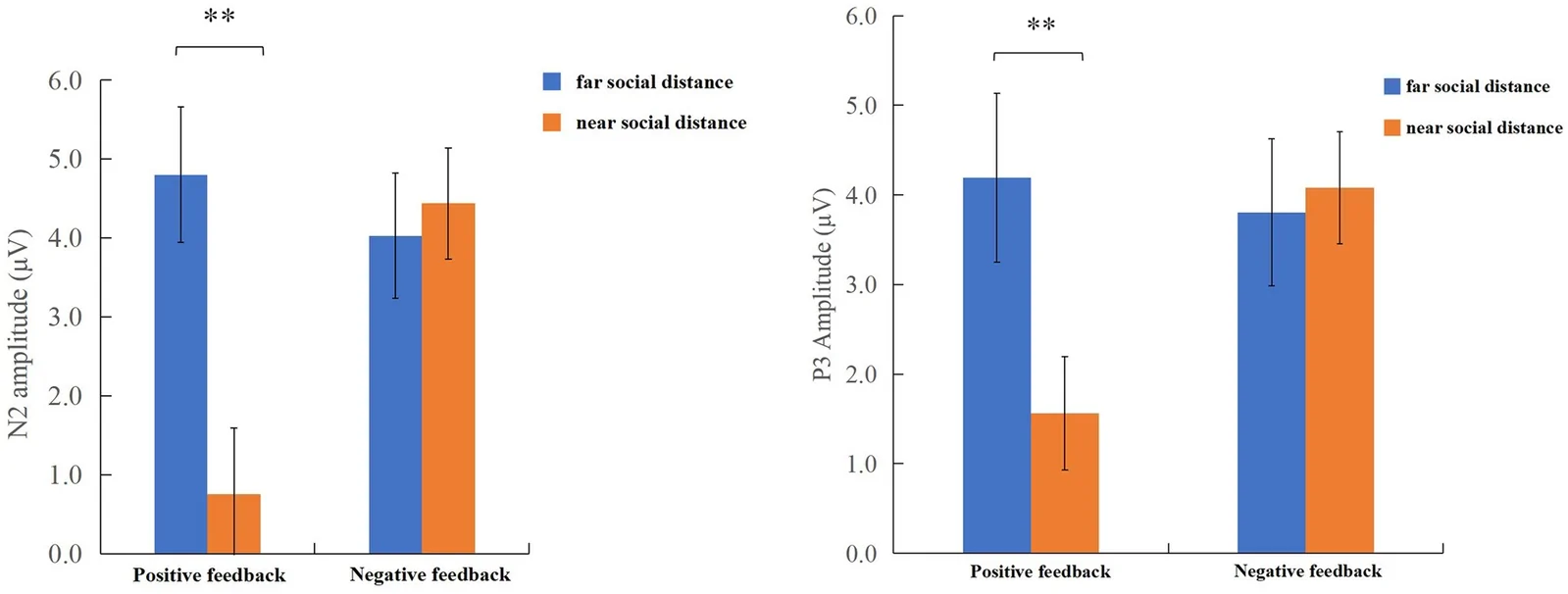
Histograms of the mean amplitude of the four conditions N2 (left), p3 components (right); error lines indicate standard errors.

##### LPP average amplitude (400–700 ms)

A 2 (feedback validity: positive vs negative) × 2 (social distance: proximity vs distance) repeated-measures ANOVA on the mean amplitude of the early components of the LPP revealed a significant main effect of feedback validity, *F* (1, 30) = 9.572, *p *< 0. 01,$\eta_{p}^{2}$ = 0.242. The amplitude was larger for negative (*M *= 6.980 ± 0.751) than for positive (*M *= 4.705 ± 0.656) feedback, 95% CI = [0.773, 3.777]. The Valence × Distance interaction was not significant (*p* = .252).

##### LPP average amplitude (701–1000 ms)

The ANOVA revealed a significant main effect of valence, *F* (1, 30) = 6.208, *p *< 0.05, $\eta_{p}^{2}$ = 0.171. The amplitude was larger for negative (*M *= 6.688 ± 0.842) than for positive (*M *= 4.472 ± 1.075), 95% CI = [0.400, 4.033]. The main effect of distance (*p*= .694) and the Valence × Distance interaction (*p*= .388) were not significant.

### Discussion

The behavioral results confirmed the key pattern from Experiment 1: positive feedback elevated implicit self-identity more than negative feedback, and this enhancement was significantly stronger when the feedback came from a socially close (vs distant) source. Negative feedback did not differ based on social distance.

EEG data revealed the time course of this effect. The N1 (100–150 ms) showed reduced negativity for positive feedback from a close source, suggesting a rapid, intuitive preference for self-enhancing in-group signals, which aligns with its role in early social cue detection (Gui *et al*. 2016). Following this, the P2 amplitude (150–250 ms) was enhanced not only for negative feedback but also for positive feedback from distant sources, indicating increased early attention to motivationally ambiguous or complex evaluations (Potts 2004). The P3 (330–430 ms) was larger overall for negative feedback, consistent with its link to enhanced processing of emotional stimuli (Ito *et al*. 1998, Bernat *et al*. 2001). Importantly, positive feedback from distant (vs close) sources also elicited a larger P3, suggesting that integrating unexpected or socially distant praise requires more effortful cognitive appraisal, which may be shaped by socio-evaluative norms (Miyamoto and Ma 2011). Finally, the early LPP (400–700 ms) showed sustained enhancement for negative feedback, reflecting prolonged and elaborative processing of socially threatening information (Kaunhoven and Dorjee 2017).

This sequence explains how social distance regulates the influence of online feedback on adolescent self-identity, making close praise more automatically reinforcing and distant or negative feedback more cognitively demanding to process.

## Discussion

This research provides empirical evidence on how evaluative feedback in digital environments impacts adolescent self-identity. By integrating the two key dimensions of online feedback—its valence (positive/negative) and the social distance of its source (near/far)—within a rigorous multi-method paradigm combining behavioral (GNAT) and neurophysiological (ERP) measures, the study elucidates the cognitive and neural mechanisms through which social evaluations shape the developing self.

### Online feedback facilitates adolescent self-identity development

The findings confirm that positive online feedback robustly enhances adolescents’ implicit self‑identity. This foundational effect aligns with Social Support Theory, which posits that positive evaluations from others (e.g. “likes”) function as salient signals of social acceptance that reinforce self‑concept (Carr *et al*. 2016). It also corroborates earlier work highlighting the role of affirmative feedback, both online and offline, in identity formation (Doran 2015) and the tendency for certain personality traits to elicit positive feedback that supports self‑development (Walther *et al*. 2011). At the neural level, negative feedback elicited larger P3 amplitudes, a pattern consistent with evidence that negative stimuli capture early attentional resources due to their motivational salience (Ito *et al*. 1998). Modulation of the LPP—a component reflecting sustained emotional and motivational processing (Kaunhoven and Dorjee 2017)—further indicated heightened attentional engagement with negative feedback (Yi‑Ping Zhong *et al*. 2014).

#### Adolescents selectively attend to in-group positive information to bolster implicit self-identity

A key contribution of this research is the demonstration that the impact of feedback is powerfully moderated by the social distance of its source, thereby integrating core principles of Social Identity Theory into the study of digital self‑formation. Behaviorally, adolescents bolstered their implicit self‑identity by preferentially weighting positive feedback from in‑group (near) sources, a hallmark of in‑group bias and favoritism (Tajfel and Turner 1979) that aligns with the concept of in‑group protection (Sedikides). Concurrently, they displayed higher implicit self‑identity scores in response to negative feedback from out‑group (far) sources, a pattern consistent with the strategy of “outgroup derogation” through which individuals mitigate self‑worth devaluation by discounting negative evaluations from distant others (Knobloch‑Westerwick 2020). Neural evidence further illuminated this strategic processing: positive feedback from a far (vs near) source elicited a larger P3 amplitude. The P3 component is linked to advanced selective attention (Lewis *et al*. 2006, Dennis 2010), top‑down emotional evaluation (Kaunhoven and Dorjee 2017), and the processing of self‑relevant information (Gray 2004). For late adolescents, this elevated P3 may signal effortful cognitive appraisal aimed at protecting self‑identity (e.g. by inhibiting the impact of negatives) or at balancing the motivational significance of positive feedback from strangers—a process that may be further nuanced by cultural norms regarding self‑evaluation and stranger interactions (Miyamoto and Ma 2011, Miyamoto and Ryff 2011).

#### Developmental specificity: the critical period of late adolescence

The study identified a clear developmental trajectory: the significant moderating effect of social distance on implicit self‑identity was specific to the late adolescent group. This finding converges with developmental perspectives that characterize late adolescence as a critical period for self‑identity transition and differentiation (Meilman, 1979). It also mirrors age‑dependent patterns in the efficacy of social support observed in other domains (Qingqi Liu *et al*. 2015), confirming that the pathway from social feedback to self‑concept is maturationally tuned. Notably, while early adolescents benefited more from positive feedback in general, and middle adolescents showed less pronounced modulation, only late adolescents exhibited the full pattern of strategic, distance‑dependent feedback processing.

#### Limitations and future directions

First, the experimental paradigm is grounded in a distinct East Asian/collectivistic cultural context, where sensitivity to evaluations from the in-group may be especially pronounced. Consequently, our sample was drawn from a specific cultural and educational context, and future research should test the generalizability of these effects. The feedback paradigms, while controlled, lack the ecological complexity of real, sustained online interactions. Future work could employ longitudinal designs and more naturalistic social media stimuli to trace the long-term development of these effects. Finally, future research could systematically examine the impact of overt social exclusion (e.g. being ignored or receiving no feedback online) on self-identity and investigate how individual differences (e.g., in social exclusion sensitivity or self-concept clarity) moderate this effect.

## Conclusion

In conclusion, this research demonstrates that adolescent self‑identity in digital environments is dynamically shaped by the interplay between what is said (valence) and who says it (social distance), with late adolescence serving as a sensitive period for this socially distanced processing. By moving beyond a monolithic view of social media’s effects, the study provides a mechanistic, developmentally grounded account of how the digital social world is actively integrated into the ongoing project of the self.

## Supplementary Material

nsag024_Supplementary_Data

## Data Availability

The datasets generated during and/or analyzed during the current study are available from the corresponding author on reasonable request.
