# Supplementary material for: An examination of the impact of online feedback and social distance on the implicit self-identity of adolescents: behavioral and ERP evidence
Source: Soc Cogn Affect Neurosci. 2026 Jun 10;21(1):nsag024. doi: 10.1093/scan/nsag024 (PMC13252597; doi:10.1093/scan/nsag024)
Supplement: nsag024_Supplementary_Data [file nsag024_supplementary_data.zip › supplementary1.docx]

**Appendix**

**Appendix 1 Self-Identity Scale (SIS)**

| **The following descriptions are descriptions of feelings and phenomena related to oneself in everyday life. Please refer to your actual situation and choose the option that best suits you.** | | | | | |
| --- | --- | --- | --- | --- | --- |
|  |  | **It doesn't apply at all** | **Some are not applicable** | **Some are applicable** | **whole**  **suit** |
| **1** | **I don't know what kind of person I am** | **○** | **○** | **○** | **○** |
| **2** | **People are always changing their opinion of me** | **○** | **○** | **○** | **○** |
| **3** | **I know how I should live** | **○** | **○** | **○** | **○** |
| **4** | **I can't be sure that something is moral or right** | **○** | **○** | **○** | **○** |
| **5** | **Most people agree on what kind of person I am** | **○** | **○** | **○** | **○** |
| **6** | **I feel like my lifestyle is very much for me** | **○** | **○** | **○** | **○** |
| **7** | **My worth is recognized by others** | **○** | **○** | **○** | **○** |
| **8** | **When I don't have acquaintances around, I feel free to be who I really am** | **○** | **○** | **○** | **○** |
| **9** | **I felt that what I was doing in my life wasn't really worth it** | **○** | **○** | **○** | **○** |
| **10** | **I feel I'm well adapted to the collective of my life** | **○** | **○** | **○** | **○** |
| **11** | **I'm proud of who I've become** | **○** | **○** | **○** | **○** |
| **12** | **People think very differently about me than I think about myself** | **○** | **○** | **○** | **○** |
| **13** | **I felt ignored** | **○** | **○** | **○** | **○** |
| **14** | **People don't seem to accept me** | **○** | **○** | **○** | **○** |
| **15** | **I changed my mind about what I wanted to get out of life** | **○** | **○** | **○** | **○** |
| **16** | **I don't know how people see me** | **○** | **○** | **○** | **○** |
| **17** | **I've changed my feelings about myself** | **○** | **○** | **○** | **○** |
| **18** | **I feel like I'm acting or doing things for utilitarian reasons** | **○** | **○** | **○** | **○** |
| **19** | **I am proud of being a member of the society in which I live** | **○** | **○** | **○** | **○** |

**Appendix 2 Scale of intensity of use of social networking sites**

| **The following are some questions about your performance when using social software (WeChat circle of friends, QQ space, etc.). Please answer according to the actual situation** | | | | | | |
| --- | --- | --- | --- | --- | --- | --- |
|  |  | **I don't agree with that at all** | **Not so much** | **indeterminacy** | **I agree** | **strongly endorse** |
| **1** | **Social software is part of my daily routine** | ○ | ○ | ○ | ○ | ○ |
| **2** | **I'm a little proud when I tell people I'm using social software** | ○ | ○ | ○ | ○ | ○ |
| **3** | **It has become my daily habit to log on to social software** | ○ | ○ | ○ | ○ | ○ |
| **4** | **When I don't log on to social media for a while, I feel disconnected from the outside world** | ○ | ○ | ○ | ○ | ○ |
| **5** | **I feel like I'm part of the social software family** | ○ | ○ | ○ | ○ | ○ |
| **6** | **I would be sad if the social software was shut down** | ○ | ○ | ○ | ○ | ○ |

**Appendix 3 Self-included scales for others**

Each picture has a pair of circles, one representing yourself and the other representing the group that gave you the thumbs up. Each pair of circles has varying degrees of overlap, which represents the social distance between you and them. Which item do you think your group's thumb-up members have with you?

**Appendix 4 GNAT task experimental material**

The study will measure subjects' implicit self-identity using the GNAT task with attribute words of self-words (I, we, own, self, myself) and non-self words (they, his, others, other's, other people, outsiders). Conceptual words were selected from the scale measuring self-identity by selecting a total of 31 self-identity words (e.g., meaningful, etc.) and non-self-identity words (future confused, etc.), after which psychology professionals were sought to conduct a seven-point rating scale (1. How high or low the vocabulary represents the self-identity of the event words. 2. How relevant the vocabulary is to the concept of self-identity), and then deleting those words that were low in relevance to the self-identity words and finally The 11 self-identification event words (*M* = 5.6, *SD* = 0.55) represented significantly greater self-identification than the 11 non-self-identification vocabulary words (*M*= 2.8, *SD* = 0.41), *t*(19) = 13.45, *d* = 5.77, *p* < 0.001.

**Appendix 5 Formula and Explanation of D Score**

The D-score, a reaction-time-invariant measure of implicit self-identity with established criterion validity (Teachman, 2007), served as the primary dependent variable. Data processing involved: (a) excluding trials with RTs < 300 ms; (b) replacing incorrect RTs with the mean correct RT of the corresponding block plus a 600-ms penalty; and (c) calculating mean RTs for the compatible (self–positive) and incompatible (self–negative) tasks. The D-score was computed as:

$$D=\frac{\overline{RT}_{Incompatible}-\overline{RT}_{Compatible}}{SD_{RT_{all}}}$$

where $\overline{RT}_{Incompatible}$​and$\overline{RT}_{Compatible}$​denote the mean RTs for the compatible and incompatible tasks, respectively, and $SD_{RT_{all}}$is the pooled standard deviation of all RTs across both tasks. Higher D-scores reflect a stronger automatic association between self-concept and positive attributes, indicating more positive implicit self-identity. Behavioral data were analyzed using a three‑factor repeated‑measures ANOVA on D‑scores in SPSS 23.0.

**A figure legend list**

**Fig. 1 Self-included scales for others**
