# Supplementary figures and images for: An examination of the impact of online feedback and social distance on the implicit self-identity of adolescents: behavioral and ERP evidence
Source: Soc Cogn Affect Neurosci. 2026 Jun 10;21(1):nsag024. doi: 10.1093/scan/nsag024 (PMC13252597; doi:10.1093/scan/nsag024)

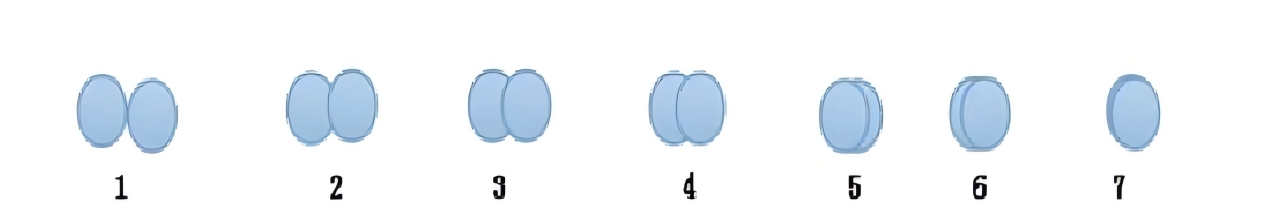

Supplement: nsag024_Supplementary_Data [file nsag024_supplementary_data.zip › supplementary 1.jpg]
